# Venous Adventitial Cystic Disease: A Review of 45 Cases Treated Since 1963

**DOI:** 10.1155/2016/5287697

**Published:** 2016-11-03

**Authors:** Corey Bascone, Mazen Iqbal, Patrick Narh-Martey, Mauricio Szuchmacher, Michael Cicchillo, Kambhampaty V. Krishnasastry

**Affiliations:** ^1^American University of Antigua, Saint George, Antigua and Barbuda; ^2^Western Reserve Health Education, Youngstown, OH, USA; ^3^Northwell Health, Manhasset, NY, USA

## Abstract

*Purpose*. To review and identify the most accurate ways of diagnosing and treating adventitial cystic disease (ACD) of the venous system.* Methods*. Cases of ACD were collected through three popular medical databases, including PubMed, Cochrane, OVID, and MEDLINE. After reviewing the literature, the sites of occurrence of 323 cases of adventitial cystic disease were documented, and all cases of arterial ACD were excluded. The clinical features, treatment, and subsequent course of 45 cases of venous ACD are included in this paper.* Results*. After reviewing all 45 cases of venous ACD , we have confirmed that the most common vessel affected is the common femoral vein, which reproduces the most common symptom of venous ACD: asymmetric lower extremity swelling worsening over time. * Conclusion*. Venous ACD most commonly affects the common femoral vein. When unilateral leg swelling occurs with or without a noticeable mass, ACD should be considered. It is best confirmed with CT venography and the treatment of choice is transluminal cyst evacuation and excision.

## 1. Introduction

Adventitial cystic disease (ACD) is an uncommon vascular anomaly that is characterized by a collection of mucinous materials in the adventitia of an affected blood vessel. These collections of materials are thought to be histologically similar to ganglia and have a propensity for extrinsically narrowing the lumen of blood vessels. This rare disease was first described in the external iliac artery in 1947 [1]. In most patients, it is commonly seen affecting the popliteal artery. This creates symptoms of claudication at a relatively young age [2]. While the majority of adventitial cystic disease cases involve the arterial system, in rare instances, these cysts can be found in the adventitia of veins. When ACD does affect the venous system, it is often mistakenly diagnosed as deep vein thrombosis. Further radiologic investigation may be essential for arriving at the correct diagnosis; however, protocols have not been established considering the rarity of the disease. Based on our review of all available literature, the main focus of this study is to conclude on the best modalities with which to successfully diagnose and treat adventitial cystic disease of the venous system.

## 2. Materials and Methods

A systematic literature search was conducted using MEDLINE, Cochrane, OVID, and PubMed databases. These search engines were utilized in aiding in the collection of all medical literature pertaining to the topic of venous adventitial cystic disease published in over fifty years. The following keywords were used: adventitial cystic disease, cystic adventitial degeneration, and venous. A total of 155 articles were found from January 1947 to March 2016, including all documented cases of ACD involving the vein, dating back to 1963. The general characteristics and disease management information of these 45 cases were then gathered and analyzed. The exclusion criterion used to formulate statistical relevance was specific to articles and/or cases pertaining to adventitial cysts of the artery.

## 3. Results

**Figure 1:**
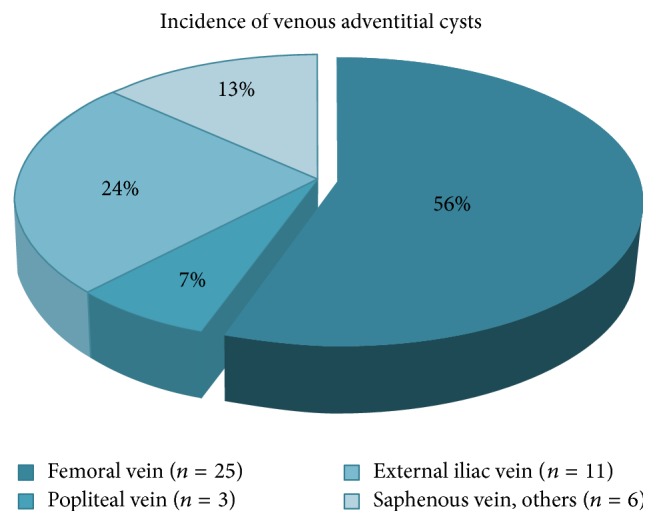


The demographical data that developed this research was comprised of this 45-case collection, in which males and females were affected equally, and the age of patients ranged anywhere from 5 to 75 years, with the mean age being 47 years. The youngest patient subject within this rather unbalanced age group involved a 5-year-old boy who is the first and only reported case of venous adventitial cystic disease in a pediatric patient [6]. Amongst the patients diagnosed with ACD of the venous system, the lower extremity was affected in 97.7% of them (in terms of symptoms or location). However, of the known cases of incidence in the world, there seems to have been a discrepancy in documented patient treatments. After a qualitative review of the literature, the discrepancy seemed to be the result of patients receiving a second procedural treatment due to recurrence, while the original treatment was documented separately: one following cyst aspiration and drainage (Johnson, 1988) and the other due to unsuccessful cyst excision (Maldonado, 1987) (Table 1). This finding alone warranted revision of the statistics regarding venous ACD, due to the fact that it seems as though revisionary treatment for two already well-documented patients was mistakenly categorized as two completely new cases. This ultimately alters our number of total patient cases worldwide but fortunately does not change anything in terms of what is recommended for treatment. With review of these forty-five cases, we found the commonly affected veins (in descending order) to be the femoral vein (25), external iliac vein (11), popliteal vein (3), small saphenous vein (2), iliofemoral vein (2), great saphenous vein (1), and the ulnar “wrist” vein (1) (Figure 1).

In order to make an accurate diagnosis, grayscale Doppler ultrasonography was found to be the best initial as well as the most convenient diagnostic test for routine clinical identification of ACD. The initial presentation warranting Doppler ultrasound consists of lower extremity swelling and/or symptoms mimicking deep vein thrombosis. Therefore, a differential diagnosis including femoral aneurysm, ganglion cyst, lipoma, venous leiomyoma, malignancy, and/or lymphadenopathy is essential for adequate patient workup [11]. CT venography remains superior to MRI and ultrasound as the most accurate diagnostic test for a venous adventitial cyst because it allows for direct observation of the cyst regardless of the level of obstruction, as well as its morphology [18].

From a treatment perspective, there were five different treatment modalities rendered in our review of the documented cases. The three treatments most commonly being carried out included cyst evacuation and excision (66.7%), cyst aspiration with drainage (13.3%), and vein resection with graft interposition (15.6%). In cases where the patient received transluminal or transadventitial evacuation followed by cyst excision, there was a recurrence rate of 20%. Patients that underwent cyst aspiration and drainage experienced an 83.3% recurrence rate and often had to follow up either with cyst excision or more successfully with vein resection and graft placement. The majority of documented cases of vein resection with graft interposition have been quite successful with a 14.3% recurrence rate. Holding a 0% recurrence rate, fenestration and drainage with sclerosis seem promising; however, there have only been two known cases where this method was utilized. With this in mind, additional data is needed to determine the reliability and curative potential of the method (Figure 3).

## 4. Discussion

Adventitial cystic disease (ACD) is rare, accounting only for 0.1% of all vascular diseases [26]. The most common vein affected in the human body is the common femoral vein. The pathogenesis of these cystic tumors gives rise to the very common clinical presentation of asymmetric lower extremity edema. In both the arterial and the venous setting, these cysts typically develop in the wall of the affected vessel, contain mucin, and result in symptoms of intermittent claudication if blood flow becomes compromised from cyst development [27]. Overexpression of the genes linked to mucin production, specifically MUC1, is associated with many types of cancer [28]. While adventitial cystic disease has yet to be linked to any form of cancer, the exact etiology of adventitial cystic disease remains controversial.

Theories behind the growth of these tumors have been linked to microtrauma, ectopic aganglionosis, systemic disorders, and even the developmental theory [29]. The theory behind the etiology of microtrauma, also known as the “Repeat Traumatic Theory,” is based on the idea that adventitia of the blood vessels undergoes cystic degeneration. This is thought to be a result of stretching and distortion of the vessel near the joints. Similarly, the idea behind systemic disorders as a cause of cyst development is also based on degeneration of the adventitia; only in this case is it due to connective tissue disease. Ectopic aganglionosis is the etiological theory that synovial cells implant into the adventitia where vessels run proximal or adjacent to the joint [29]. The development theory is technically based on the same idea; only in this situation do the mesenchymal cells implant during human embryological development.

While all of these theories are generally accepted as ways in which cystic tumors develop in the venous system, it is still unclear whether a developmental difference exists between cysts of the vein and cysts of the artery. This is simply because there are not enough cases of venous adventitial cystic disease to derive conclusions from. In 1998, Levien and Benn identified 323 confirmed cases of ACD of the blood vessels, only 17 (5.3%) of which involved the vein [30]. This small number of reported cases may be due not only to the diseases low incidence, but also to the difficulty in making the proper diagnosis, as ACD can also be commonly confused with a hip joint synovial cyst.

**Figure 2:**
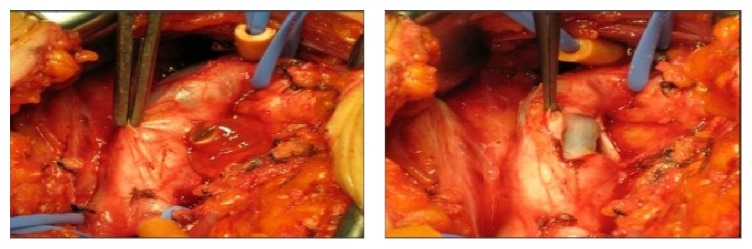
Operative image shows the gelatinous material in a large cyst arising in the lateral wall of the common femoral vein and compressing the lumen [3].

**Figure 3:**
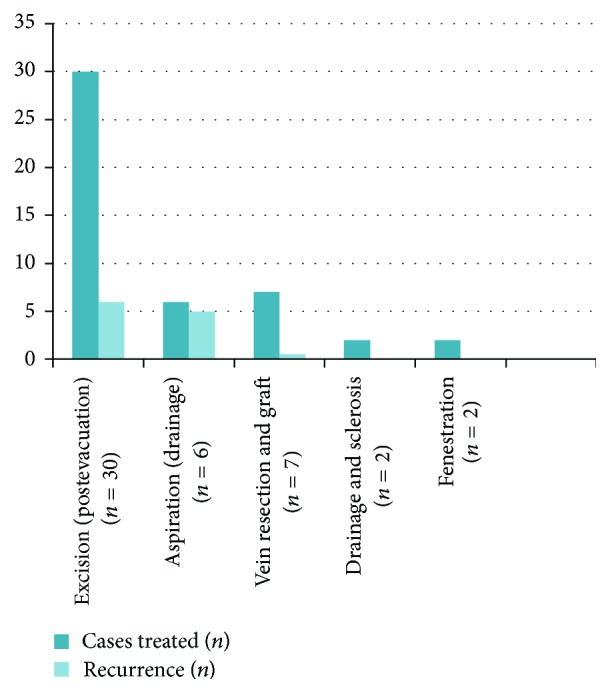


**Figure 4:**
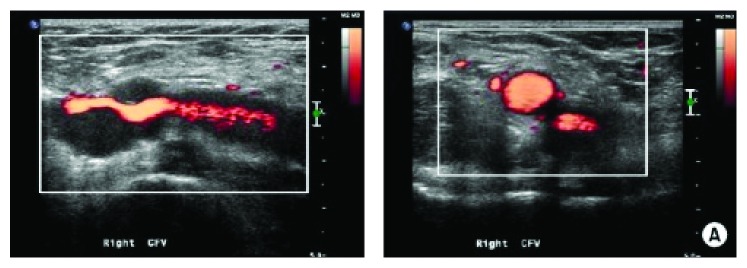
Ultrasound imaging shows a typical hypoechoic fluid-filled cyst with a posterior acoustic window [3].

Diagnosis of an adventitial cyst involves a thorough history and physical exam, as well as necessary imaging modalities. Traditionally, ultrasound and venography were commonly employed as the best way to make a diagnosis. If ultrasound is used in visualizing a cyst, it may show a hypoechoic fluid-filled cyst, accompanied with a posterior acoustic window [5] (Figure 4). The MRI findings of adventitial cystic disease are quite clear as cysts, typically appearing as regions of homogeneous low signal intensity on T1-weighted images and of multiloculated high signal intensity adjacent to vessels on T2-weighted images [31]. Performing CT venography in patients with venous adventitial cystic disease can reveal the site and the extent of the obstruction, and it may show a classic scalloped appearance or hourglass narrowing caused by the extrinsic compression of the vessel lumen [18] (Figure 2). Although all of these diagnostic methods are useful in identifying these cystic tumors, CT venography remains the superior diagnostic test because it allows for direct observation of the cyst, regardless of the level of obstruction [18] (Figure 6). In addition, CT venography has the ability to display collateral vasculature in relation to the cystic lesion, as well as possible communication between a cyst and an adjacent joint.

In both adventitial cystic diseases of the arterial system and of the venous system, cysts have a tendency to recur after treatment [18]. Minimally invasive management has been reported with image-guided needle aspiration of adventitial cysts, but incomplete evacuation of cysts secondary to high viscosity and the mucin-secreting mesenchymal cells that are left in situ had resulted in high recurrence rates [6, 11]. Endovascular treatment is also ineffective because it fails to deal with the underlying cause of compression and may become problematic with stent's crossing joint lines and with thrombosis in low flow veins [19]. Therefore, it is essential for physicians to utilize surgical intervention as the treatment of choice.

If the vein of the patient is intact and without compromise to the lumen, transluminal or transadventitial evacuation of the cyst prior to cyst wall excision is performed. With a 20% recurrence rate, care should be taken by the operative surgeon to ensure a thorough wall resection. Without a proper wall excision, mesenchymal cells of the mucoid cyst may be left behind in the adventitia and secrete enough mucin to reoccur.

Vein resection with graft placement of either the great saphenous or small saphenous vein has a 14.3% recurrence rate. However, it is noted that this method should only be used if the vein being treated is compromised in any way or if the surgeon is not able to adequately visualize and operate on the blood vessel [13]. It may be beneficial before both cyst excision and vein resection to perform three-dimensional CT reconstruction (using volume rendering or MRA) for optimal preoperative planning [31]. Based on our review, further follow-ups with the patient postoperatively have also been essential in reliably producing a successful outcome.

## 5. Conclusion

**Figure 5:**
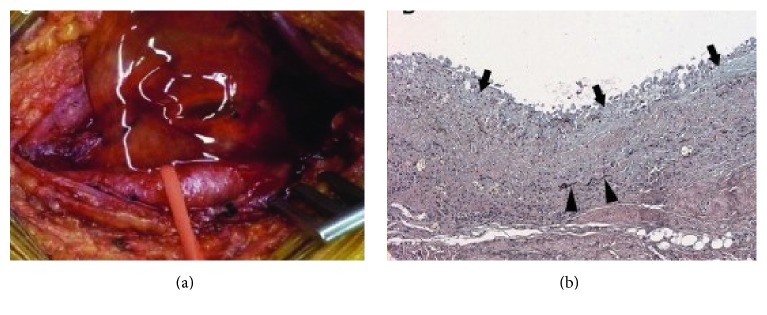
(a) Thick gelatinous material issued from the cystic cavity close to the external iliac vein in operative finding. (b) Pathology reveals a cystic wall composed of fibrous tissue with increased proteoglycans (arrows) and few elastic fibers (arrowheads) [4].

**Figure 6:**
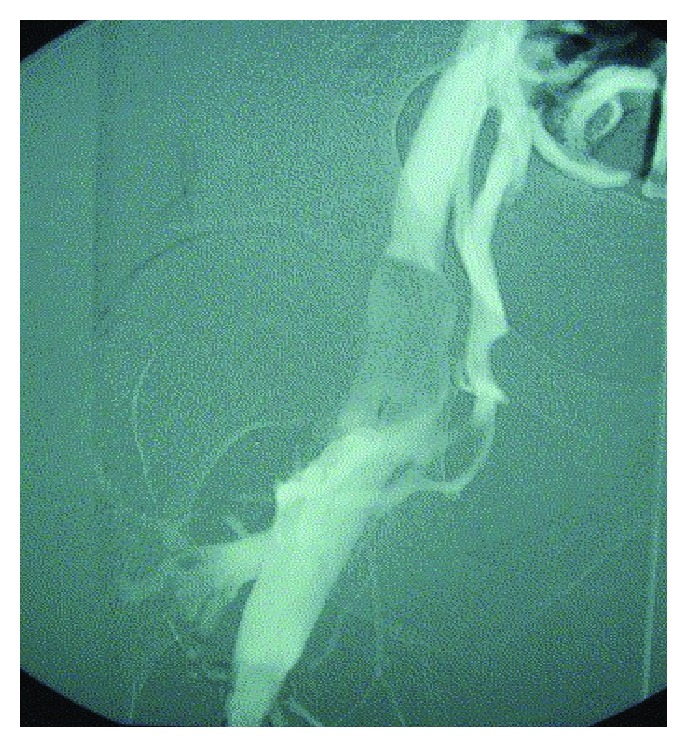
Ascending venogram shows obstruction to flow in the CFV caused by an extrinsic mass with typical scalloping of the lumen caused by cystic adventitial disease [5].

Adventitial cystic disease of the venous system is a rare occurrence with only a few reported cases in the world's literature. Its pathogenesis has been poorly understood and clinical presentation mimics DVT. Differential diagnosis including synovial cyst, aneurysm, and malignancy is essential due to different operative strategies. In general, endovascular and minimally invasive treatment had higher recurrences, thus making transadventitial or transluminal evacuation of the mucoid cysts with removal of the cystic wall the preferred surgical intervention (Figure 5). When strong clinical suspicion exists, physicians should pursue advanced radiological studies to identify these cysts.

## Figures and Tables

**Table 1:** Venous adventitial cystic disease demographics.

Author and year	Gender, age	Physical signs, DVT symptoms (presence)	Location	Treatments used (initial, final)	Recurrence (resolution)
Mentha, Switzerland, 1963	FM, 30 yo	Mass (0)	SSV (1)	CA (1)	1
Gomez Ferrer, Spain, 1966	M, 43 yo	Inguinal mass (1)	CFV (1)	CE (1)	0
Lavarde, France, 1972	M, 43 yo	Pain (0)	PV (1)	CE (1)	0
Leu et al., Switzerland, 1977	FM, 29 yo	Mass (0)	UV (1)	CA (1)	0
Matsubara et al., 1978	M, 57 yo	(1)	EIV (1)	CE (1)	0
Chafke et al., 1977	M, 57 yo	(1)	EIV (1)	CE (1)	0
Frileux et al., 1979	FM, 40 yo	(1)	EIV (1)	CE (1)	0
Fyfe et al., 1980	M, 42 yo	Inguinal mass (0)	CFV (1)	CE (1)	1
Annets and Graham, 1980	FM, 23 yo	Inguinal mass (1)	CFV (1)	CE (1)	1
Matsubara et al., 1982	M, 48 yo	(1)	CFV (1)	CE (1)	0
Ohta et al., 1984	M, 48 yo	Inguinal mass (1)	EIV (1)	CE (1)	0
Ito et al., 1984	FM, 55 yo	(1)	EIV (1)	F (1)	0
O'Neill et al., 1987	M, 61 yo	(1)	CFV (1)	CE (1)	0
Lie et al., 1991	M, 40 yo	Mass (0)	SSV (1)	CA (1)	1
Paty et al., 1992	M, 65 yo	(1)	CFV (1)	CA (1)	1
Schraverus et al., 1997 [7]	M, 56 yo	(1)	PV (1)	VR (1)	1
Desjardins et al., 1997 [8]	FM, 32 yo	(1)	CFV (1)	CE (1)	1
Yoshii et al., 1998 [9]	FM, 75 yo	Mass (0)	GSV (1)	CA (1)	1
Maldonado et al., 2001	FM, 56 yo	Inguinal mass (1)	EIV (1)	CE (1)	1
Fukui et al., 2004 [10]	FM, 32 yo	(1)	CFV (1)	CE (1)	0
Gasparis et al., 2004 [11]	M, 37 yo	(1)	IF (1)	TL (1), VR (1)	0
Maldonado-Fernández, 2004 [12]	FM, 56 yo	(1)	EIV (1)	CE (1)	1 (CE)
Sugimoto et al., 2004 [13]	FM, 48 yo	(1)	CFV (1)	VR (1)	0
Cho and Shin, 2005 [14]	M, 52 yo	(1)	CFV (1)	CE (1)	0
Dix et al., 2006 [5]	M, 28 yo	(1)	CFV (1)	TL (1), CE (1)	0
Sakamoto et al., 2006 [15]	FM, 56 yo	(1)	PV (1)	CE (1)	0
Desjardins et al., 1997 [8]	FM, 48 yo	(1)	CFV (1)	CE (1)	0
	M, 61 yo	(1)	CFV (1)	CE (1)	0
Kohno et al., 2007 [16]	FM, 48 yo	(1)	CFV (1)	F (1)	0
Zhang et al., 2008 [17]	M, 54 yo	(1)	EIV (1)	CE (1)	0
Seo et al., 2009 [18]	M, 69 yo	(1)	CFV (1)	VR (1)	0
Johnson et al., 2009 [19]	M, 66 yo	(1)	CFV (1)	CA (1), DS (1)	1 (DS)
Morizumi et al., 2010 [20]	M, 28 yo	(1)	CFV (1)	CE (1)	0
Jayaraj et al., 2011 [21]	M, 36 yo	(1)	CFV (1)	TL (1), VR (1)	0
Kwun and Suh, 2011 (2 cases) [3]	FM, 54 yo	(1)	CFV (1)	CE (1)	0
	FM, N/A	(1)	CFV (1)	CE (1)	0
Jones et al., 2012 [6]	M, 5 yo	(1)	CFV (1)	CE (1)	0
Park et al., 2013 [4]	FM, 50 yo	(1)	EIV (1)	CE (1)	0
	FM, 32 yo	(1)	EIV (1)	VR (1)	0
Michaelides et al., 2014 [22]	FM, 51 yo	(1)	IF (1)	VR (1)	0
Ann et al., 2015 [23]	M, 70 yo	(1)	EIV (1)	CE (1), DS (1)	1 (DS)
Chen et al., 2015 [24]	M, 58 yo	(1)	CFV (1)	CE (1)	0
	FM, 38 yo	(1)	CFV (1)	CE (1)	0
	M, 47 yo	(1)	CFV (1)	CE (1)	0
O'Loghlen et al., 2016 [25]	M, 31 yo	(1)	CFV (1)	CE (1)	0

CFV: common femoral vein; EIV: external iliac vein; PV: popliteal vein; GSV: great saphenous vein; IF: iliofemoral; SSV: small saphenous vein; UV: ulnar vein (wrist); N/A: not available; (*n*): number of patients.

Symptomology: (0) = indicative of patient lacking the presence of the DVT symptoms/swelling; (1) = presence of DVT symptoms/swelling.

CE: cyst excision/resection (postevacuation); CA: cyst aspiration and drainage; VR: vein resection (with graft placement); F: fenestration; TL: thrombolysis; DS: drainage and sclerosis.
